# Olanzapine and haloperidol for the treatment of acute symptoms of mental disorders induced by amphetamine-type stimulants: A randomized controlled trial

**DOI:** 10.1097/MD.0000000000009786

**Published:** 2018-02-23

**Authors:** Xiaobin Xue, Yun Song, Xiaojie Yu, Qiang Fan, Jiyou Tang, Xu Chen

**Affiliations:** a Department of Substance Abuse, Qingdao Mental Health Center, Qingdao; b Department of Neurology, Qianfoshan Hospital Affiliated of Shandong University, Jinan; c Department of Psychiatry Nursing, Qingdao Mental Health Center, Qingdao; d Department of Substance Abuse, Ningbo An Kang Hospital, Fenghua; e Department of Substance Abuse, Shandong Mental Health Center, Jinan, China.

**Keywords:** amphetamine-type stimulants, haloperidol, mental disorder, olanzapine

## Abstract

**Background:**

This study aimed to compare olanzapine and haloperidol efficacies in the treatment of acute psychiatric symptoms due to amphetamine-type stimulants (ATSs).

**Methods:**

The Zelen II design method was used; 124 patients with acute mental disorders due to amphetamine were randomly divided into olanzapine group (n = 63) and haloperidol group (n = 61). Then, a 4-week open-label medical therapy was performed. Clinical Global Impression Scale Item 2 was employed to evaluate the onset time; meanwhile, Brief Psychiatric Rating Scale (BPRS) was used at baseline and at posttreatment weeks 1, 2, and 4. Moreover, adverse reactions during the treatment were recorded.

**Results:**

Onset time in the olanzapine group was significantly earlier than in the haloperidol group; BPRS scores in the olanzapine group were significantly lower than haloperidol group values at 1 and 2 weeks of treatment. The overall effective rates had no statistically significant difference.

**Conclusion:**

Short-term olanzapine and haloperidol treatments had equivalent efficacies in the treatment of acute symptoms of mental disorders due to ATSs; however, olanzapine administration resulted in relatively earlier disease onset, with less adverse reactions.

## 1 Introduction

With economic development in China, new drugs related to traditional drugs, such as opium and heroin, emerge in an endless stream. Due to the characteristics of new drugs, including relatively low physical dependence, severe psychological dependence, and strong hallucinogenic and excitatory effects, there is a tendency that they would replace traditional drugs. The monitoring data on domestic drug abuse in 2014 showed that amphetamines count among the most important drugs, accounting for about 36.8%, and top new drugs abused, accounting for 70.5%.^[1]^ Harms due to amphetamines show a high incidence trend^[2]^; the induced mental disorders not only result in personal health damage, but also affect social security, such as “drug driving.” The common symptoms of mental disorders due to amphetamine include agitation, aggressive behavior, delusions, hallucinations, anxiety, and depression.^[3,4]^

Currently, treatment of mental disorders due to amphetamines is generally symptomatic, with no potent medication available. In China, the revised version of “The guidelines of Amphetamine Drug Dependent Diagnosis and Treatment” published in 2009 clearly proposed that antipsychotics could be used to treat psychiatric symptoms, pointing out that psychological treatment is a therapeutic option.^[5]^ Meanwhile, studies found that repeated transcranial magnetic stimulation and electrical shock can also be used for the treatment of mental disorders.^[6,7]^ A review by Glasner-Edwards describes in great detail the important role of antipsychotics in the treatment of psychiatric symptoms due to amphetamines, while emphasizing the importance of psychotherapy and social rehabilitation.^[2]^

The treatment principle of the Chinese guidelines clearly points out that olanzapine and haloperidol can be used when psychotic symptoms, such as hallucinations, delusion, excitement, and agitation, occur in patients abusing amphetamine.^[5]^ Relevant clinical studies both in China and abroad have confirmed that atypical antipsychotics, including olanzapine, aripiprazole, risperidone, quetiapine, and clozapine, show better efficacy in the treatment of mental symptoms.^[3,8–12]^ Indeed, Leelahanaj et al^[8]^ found that olanzapine and haloperidol have better therapeutic effects on psychotic symptoms during a 4-week observation; Yap et al^[9]^ also confirmed that olanzapine has higher effects in emergency treatment for controlling excitatory symptoms. Relevant basic studies demonstrated that olanzapine has neuroprotective and therapeutic effects in an amphetamine-mediated psychotic animal model.^[13,14]^

This study aimed to compare the onset times and therapeutic effects of olanzapine and haloperidol in the treatment of psychosis induced by ATSs, enriching clinical treatment experience.

## 2 Subjects and methods

### 2.1 Subjects

Patients hospitalized from January 2013 to December 2013 in the hospital were enrolled as study subjects. Inclusion criteria were: age between 18 and 60 years, with positive methamphetamine urine test; meeting the diagnostic criteria for mental disorders due to ATSs as defined by the American Society for the Diagnosis and Statistics of Mental Disorders (4th edition) (DSM-IV); Brief Psychiatric Rating Scale (BPRS) > 35 points at baseline; and signed informed consent (by the patients or their guardians). Exclusion criteria were: dependence on other nonamphetamine-type psychoactive substances; suicidal behavior or serious suicidal tendency; combined with serious physical illness; a history of mental illness; serious impulse and self-injury behaviors; antipsychotic treatment prior to enrollment; allergic reactions to olanzapine or haloperidol; and pregnancy or lactation in women. Treatment termination criteria were: serious adverse reactions; informed consent withdrawal; abnormalities in laboratory or specific examinations, with the clinician deciding the unsuitability of the case to continue; lack of treatment response or disease aggravation after 2 weeks of treatment; and suicide thoughts or other serious diseases. This study was reviewed and approved by the ethics committee of the participating units. Informed consent was obtained from all individual participants included in the study.

Complete data for a total of 124 patients were collected, including 63 cases in the olanzapine group (42 males and 21 females), and 61 in the haloperidol group (44 males and 17 females). There was no statistically significant difference in gender composition between the 2 groups (χ^2^ = 0.44, *P* > .05). Average ages in the olanzapine and haloperidol groups were 29.5 ± 5.2 and 31.2 ± 6.8 years, respectively; mean disease courses were 16.4 ± 3.9 and 17.1 ± 5.1 months, respectively, indicating no statistically significant difference (*t* = 1.29, 1.10, *P* > .05).

## 3 Methods

### 3.1 Design

**Figure 1:**
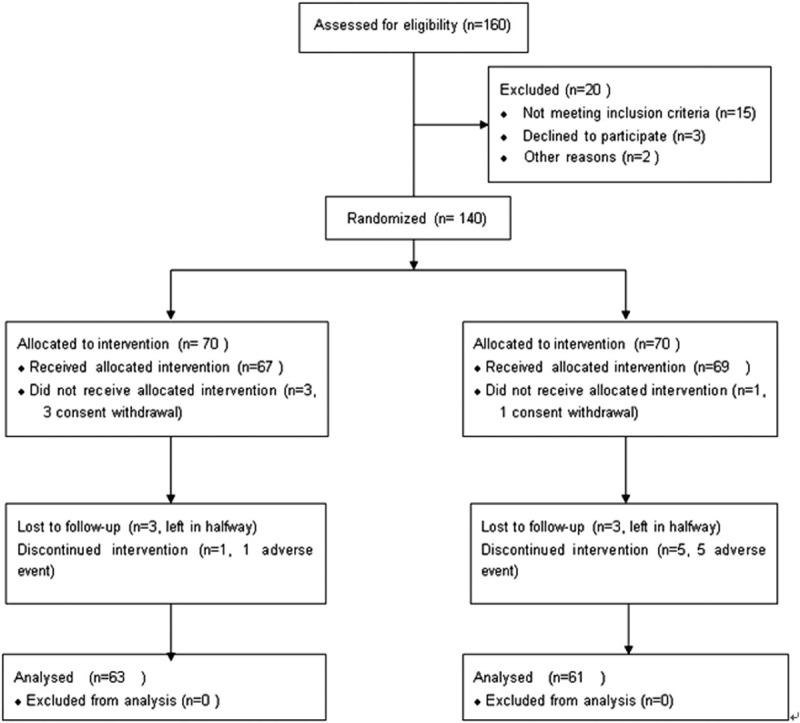
Flow of participates through the trial.

In this study, the Zelen II design method was used.^[15]^ Through drawing from previous study results,^[10,11]^ the EpiCalc2000 software was used to preliminarily estimate the sample size of no less than 108 cases. Consecutive subjects who met the inclusion criteria were assigned to groups based on a randomized number table. Then, consent for olanzapine therapy was obtained from enrolled subjects of the study group. The medication is open-label. The flow of participates through this trial was shown in Fig. 1.

### 3.2 Medication

The study group was administered olanzapine therapy; the initial dose was 5 mg/day, increased to 10 mg/day at 7 days, with a dose range of 5 to 20 mg/day. The control group received haloperidol therapy, with an initial dose of 2 mg/day, which was increased to 5 mg/day at 7 days, with a dose range of 2 to 12 mg/day. The dose was gradually increased according to the tolerance of patients during the treatment period; the dose could be adjusted at any time. Moreover, the patients were administered twice daily (noon and night). The patients were not treated with antipsychotics, antidepressants, mood stabilizers, and nonspecific anxiolytics. Clonazepam at 1 to 4 mg/day was coadministered at night with sleep disorder not improving; benzhexol at 2 to 6 mg/day was supplemented in case of extrapyramidal reactions; propranolol at 20 to 60 mg/day was used for tachycardia or akathisia; and vitamin B6 at 30 to 60 mg/day should be added for gastrointestinal reactions.

### 3.3 Evaluation of efficacy and adverse drug reactions

The therapeutic effects were evaluated by BPRS. Including of 5 factors (anxiety and depression, lake of vitality, thinking problems, activation, and hostile suspicion), BPRS was used to assess the psychopathological symptoms by psychiatrists who finished the training and passed the consistency check with a satisfied result. On the basis of the psychiatrists’ observations and the patients’ statements, 18 items with scores ranging from 0(absent) to 7(most severe) were scaled and valued. Efficacy was determined by the rate of BPRS reduction 4 weeks after treatment, and reduction rate ≥60% reflected a significant effect; reduction rate ≥30% was considered to indicate effectiveness. Clinical Global Impression Scale Item (CGI-SI) 2 was used to evaluate the onset time, as follows: 0, no evaluation; 1, very obvious improvement; 2, obvious improvement; 3, improvement; 4, no changes; 5, slight degradation; 6, obvious degradation; and 7, very obvious degradation. All evaluations were performed based on the changes of clinical symptoms. Onset time was assessed by the required time to achieve CGI-SI evaluation as “improvement.” Serious adverse events and abnormal results in auxiliary examinations with clinical significance were timely recorded, and their correlations with medicine assessed by clinicians. For patients with abnormal auxiliary examination results but without clinical significance, or those with abnormal auxiliary examination results and clinical significance but with approval from guardians, treatment could be continued. Evaluations were performed by 6 psychiatrists with training regarding the experimental procedure and scale. At the end of the training, the consistency of BPRS score was *r* = 0.92; that of CGI-SI was K_w_ = 0.89.

### 3.4 Statistical methods

SPSS14.0 was used for analyses. General data were assessed by Chi-square test; group-wise comparison was performed by independent samples *t* test; intergroup comparison used *t* test for matching samples. Determination of normality and variance homogeneity was performed before *t* test; nonparametric test was performed for data with heterogeneity of variance. Data of the patients who did not receive olanzapine therapy in the study group were included. *P* < .05 was considered statistically significant.

## 4 Results

### 4.1 Efficacy

The average onset time was 6.31 ± 1.74 and 9.42 ± 2.08 days in the study and control groups, in the acute phase (within 4 weeks), respectively. Onset occurred in the study group before controls (Z = 4.31, *P* < .05). At 4 weeks after treatment, total effective rates (BPRS reduction ≥30%) were 96.1% and 91.5%, in the study and control groups, respectively; the difference between the 2 groups was not statistically significant (χ^2^ = 1.12, *P* > .05).

### 4.2 BPRS scores before and after treatment

Total BPRS scores and scores of various factors at 1, 2, and 4 weeks after treatment in the study group decreased compared with baseline values, with statistically significant differences (*P* < .05).

In the control group, the score of activation factors at 1 week after treatment decreased compared with baseline (*P* < .05). Except the lack of activation factors, total BPRS scores and scores of various factors at 2 weeks after treatment decreased compared with baseline values (*P* < .05). The total scores and those of various factors at 4 weeks after treatment decreased compared with baseline values (*P* < .05).

**Figure 2:**
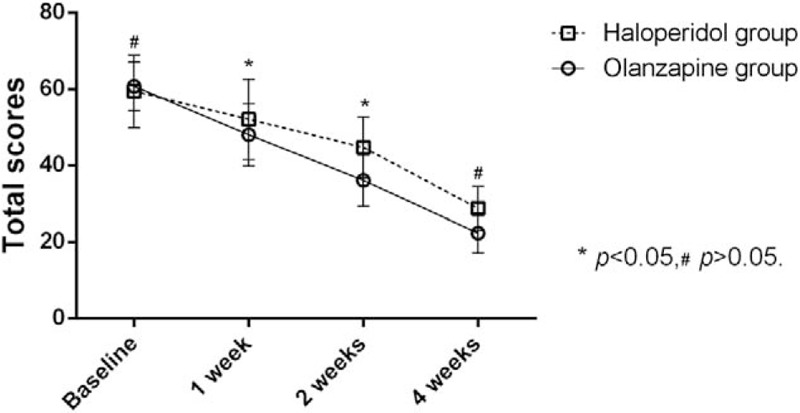
Comparison of Brief Psychiatric Rating Scale (BPRS) total scores between olanzapine group and halperidol group, there are significant difference at the end of 2 and 4 weeks (*P* < .05), but no difference at the terminal (*P* > .05).

Group-wise comparison found that the total BPRS score and scores of anxiety and depression, as well as the lack of activation factors at 1 and 2 weeks in the study group were all reduced compared with control values (*P* < .05). At the end of week 4, scores of anxiety and depression, lack of activation factors and thinking disorders were lower in the study group than control values (*P* < .05) (Table 1). Changes of total BPRS scores are shown in Fig. 2.

**Table 1:**
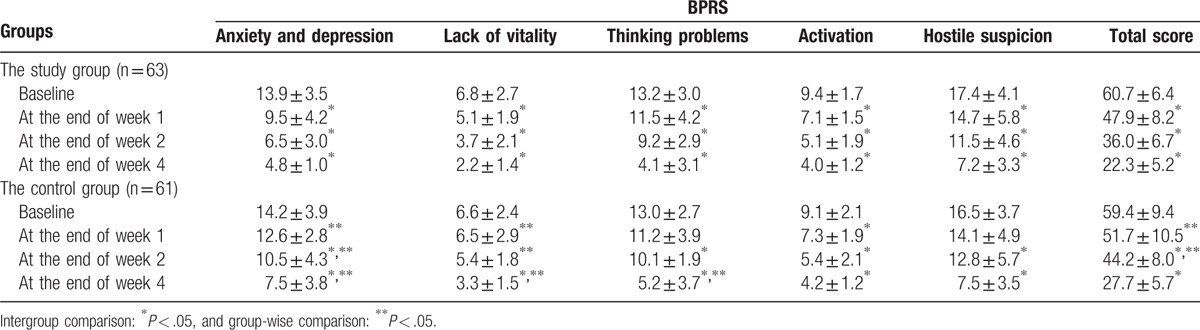
Brief Psychiatric Rating Scale (BPRS) scores at different time points in the study and control groups.

### 4.3 Adverse reactions of antipsychotics

The data are summarized in Table 2. During the 4-week treatment period, there was no serious adverse drug reaction in the 2 groups. However, the incidence rate of adverse drug reactions in the study group was comparatively lower than that in the control group, with a statistically significant difference (*P* < .01).

**Table 2:**
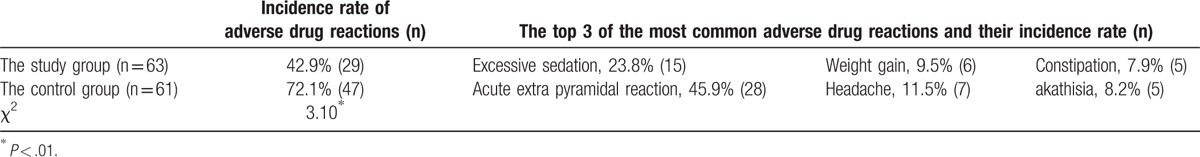
Adverse drug reactions in the study and control groups.

## 5 Discussion

This study found that olanzapine and haloperidol effectively improved psychotic symptoms due to amphetamines in a short term. The onset time of olanzapine was earlier, with lower incidence of adverse drug reactions compared with haloperidol values. Meanwhile, olanzapine was superior in improving anxiety and depression, lack of vitality, and thinking disorders.

Mental disorders due to ATSs are widespread in the abuse population with psychotic symptoms as the main concern.^[16]^ ATS has broad effects on the central nervous system, which has an effective role in the dopamine (DA) system.^[17]^ ATS may confer to drug abusers a sense of strong pleasure by increasing the release of DA in some areas of the cerebrum; meanwhile, DA dysfunction in long-term abusers may directly cause other psychotic symptoms.^[17,18]^ In the clinical diagnosis and treatment of ATS abusers, studies^[19,20]^ have revealed that ATS drugs produce effects similar to schizophrenia. In addition, ATS has significant interference on the 5-HT system in the brain.^[21]^ Mood changes in patients with acute poisoning or long-term smokers are correlated with 5-HT dysfunction. Relevant basic research^[22]^ has demonstrated that ATS causes anxiety and depression, emotional indifference in humans, and the corresponding behavioral changes in animals.

Olanzapine is used as an atypical antipsychotic; its functional receptors are mainly 5-HT2 and D2 receptors, and its affinity to 5-HT2 is greater than that to D2. Related studies found that olanzapine is more effective in schizophrenia patients with anxiety and depression.^[23]^ This study also confirmed that olanzapine had better effects in improving emotional symptoms such as anxiety and depression at the early stage of treatment. These effects were better than those of haloperidol, which may be related to the 5-HT receptor. Olanzapine more pronouncedly improves psychotic symptoms due to ATS, which is consistent with previous findings.^[8,24]^ Moreover, it exerted good antipsychotic effects with few extrapyramidal adverse reactions and better adherence; this may be related to the affinity of D2 receptor of 60% to 80%.^[25]^ Meanwhile, blockade of olanzapine to M and H1 receptors may explain the presence of excessive sedation and constipation.

Haloperidol is a D_2_ receptor blocker with simple action. This study found that haloperidol was ideal in improving psychotic symptoms, but did not significantly improve anxiety and depression, which may be related to its simple action on D_2_ receptor. However, with the improvement of psychotic symptoms, clinical signs secondary to psychotic symptoms were gradually alleviated. This difference with olanzapine may be related to the singularity of its receptor. Meanwhile, we also found that early beneficial effects of olanzapine on factors such as anxiety and depression may be correlated with its impact on the 5-HT2A receptor.

In addition, adverse reactions of olanzapine were significantly fewer than those of haloperidol, which made olanzapine more easily accepted by the patients despite related excessive sedation and weight gain.

This study did not include more detailed and quantified horizontal comparisons for medical doses and degrees of adverse reactions; meanwhile, this trial was also limited by the short observation time. These limitations will be addressed and improved in future studies.

## References

[1] http://www.sda.gov.cn/WS01/CL0051/128740.html. (Accessed September 8, 2015).

[2] Glasner-Edwards S Mooney LJ Methamphetamine psychosis: epidemiology and management. CNS Drugs 2014;28:1115–26. 25373627 10.1007/s40263-014-0209-8 PMC5027896

[3] Zarrabi H Khalkhali M Hamidi A Clinical features, course and treatment of methamphetamine-induced psychosis in psychiatric inpatients. BMC Psychiatry 2016;16:44. 26911516 10.1186/s12888-016-0745-5 PMC4766712

[4] Pasic J Russo JE Ries RK Methamphetamine users in the psychiatric emergency services: a case-control study. Am J Drug Alcohol Abuse 2007;33:675–86. 17891660 10.1080/00952990701522732

[5] http://www.nhfpc.gov.cn/zwgkzt/wsbysj/200912/45283.shtml. (Accessed January 11, 2010).

[6] Gorelick DA Zangen A George MS Transcranial magnetic stimulation in the treatment of substance addiction. Ann N Y Acad Sci 2014;1327:79–93. 25069523 10.1111/nyas.12479 PMC4206564

[7] Ziaaddini H Roohbakhsh T Nakhaee N Effectiveness of electroconvulsive therapy in persistent methamphetamine psychosis: a pilot study. Addict Health 2015;7:14–23. 26322206 PMC4530189

[8] Leelahanaj T Kongsakon R Netrakom P A 4-week, double-blind comparison of olanzapine with haloperidol in the treatment of amphetamine psychosis. J Med Assoc Thai 2005;88 suppl 3:S43–52. 16858942

[9] Yap CY Taylor DM Knott JC Intravenous midazolam-droperidol combination, droperidol or olanzapine monotherapy for methamphetamine-related acute agitation: subgroup analysis of a randomized controlled trial. Addiction 2017;112:1262–9. 28160494 10.1111/add.13780

[10] Verachai V Rukngan W Chawanakrasaesin K Treatment of methamphetamine-induced psychosis: a double-blind randomized controlled trial comparing haloperidol and quetiapine. Psychopharmacology (Berl) 2014;231:3099–108. 24535654 10.1007/s00213-014-3485-6

[11] Farnia V Shakeri J Tatari F Randomized controlled trial of aripiprazole versus risperidone for the treatment of amphetamine-induced psychosis. Am J Drug Alcohol Abuse 2014;40:10–5. 24359506 10.3109/00952990.2013.861843

[12] Seddigh R Keshavarz-Akhlaghi AA Shariati B Treating methamphetamine-induced resistant psychosis with clozapine. Case Rep Psychiatry 2014;2014:845145. 25530901 10.1155/2014/845145 PMC4228824

[13] He J Xu H Yang Y Neuroprotective effects of olanzapine on methamphetamine-induced neurotoxicity are associated with an inhibition of hyperthermia and prevention of Bcl-2 decrease in rats. Brain Res 2004;1018:186–92. 15276877 10.1016/j.brainres.2004.05.060

[14] Abekawa T Ito K Nakagawa S Olanzapine and risperidone block a high dose of methamphetamine-induced schizophrenia-like behavioral abnormalities and accompanied apoptosis in the medial prefrontal cortex. Schizophr Res 2008;101:84–94. 18262394 10.1016/j.schres.2007.12.488

[15] Zelen M A new design for randomized clinical trials. N Engl J Med 1979;300:1242–5. 431682 10.1056/NEJM197905313002203

[16] Glasner-Edwards S Mooney LJ Marinelli-Casey P Clinical course and outcomes of methamphetamine-dependent adults with psychosis. J Subst Abuse Treat 2008;35:445–50. 18294802 10.1016/j.jsat.2007.12.004

[17] Miyazaki M Noda Y Mouri A Role of convergent activation of glutamatergic and dopaminergic systems in the nucleus accumbens in the development of methamphetamine psychosis and dependence. Int J Neuropsychopharmacol 2013;16:1341–50. 23195702 10.1017/S1461145712001356

[18] Anneken JH Angoa-Perez M Kuhn DM 3,4-Methylenedioxypyrovalerone prevents while methylone enhances methamphetamine-induced damage to dopamine nerve endings: beta-ketoamphetamine modulation of neurotoxicity by the dopamine transporter. J Neurochem 2015;133:211–22. 25626880 10.1111/jnc.13048 PMC4759647

[19] Paterson NE Vocci F Sevak RJ Dopamine D3 receptors as a therapeutic target for methamphetamine dependence. Am J Drug Alcohol Abuse 2014;40:1–9. 24359505 10.3109/00952990.2013.858723

[20] Hsieh JH Stein DJ Howells FM The neurobiology of methamphetamine induced psychosis. Front Hum Neurosci 2014;8:537. 25100979 10.3389/fnhum.2014.00537 PMC4105632

[21] Chiu HY Chan MH Lee MY Long-lasting alterations in 5-HT2A receptor after a binge regimen of methamphetamine in mice. Int J Neuropsychopharmacol 2014;17:1647–58. 24763081 10.1017/S1461145714000455

[22] Jang CG Whitfield T Schulteis G A dysphoric-like state during early withdrawal from extended access to methamphetamine self-administration in rats. Psychopharmacology (Berl) 2013;225:753–63. 23007601 10.1007/s00213-012-2864-0 PMC3547144

[23] Hershenberg R Gros DF Brawman-Mintzer O Role of atypical antipsychotics in the treatment of generalized anxiety disorder. CNS Drugs 2014;28:519–33. 24794100 10.1007/s40263-014-0162-6

[24] Misra LK Kofoed L Oesterheld JR Olanzapine treatment of methamphetamine psychosis. J Clin Psychopharmacol 2000;20:393–4. 10831035 10.1097/00004714-200006000-00023

[25] Raedler TJ Knable MB Lafargue T In vivo determination of striatal dopamine D2 receptor occupancy in patients treated with olanzapine. Psychiatry Res 1999;90:81–90. 10482380 10.1016/s0925-4927(99)00010-4

